# Methyl 7-meth­oxy-9-oxo-9*H*-xanthene-2-carboxyl­ate

**DOI:** 10.1107/S1600536809003602

**Published:** 2009-02-06

**Authors:** Paweł Niedziałkowski, Tadeusz Ossowski, Artur Sikorski

**Affiliations:** a University of Gdańsk, Faculty of Chemistry, Sobieskiego 18/19, 80-952 Gdańsk, Poland

## Abstract

The crystal structure of the title compound, C_16_H_12_O_5_, is stabilized by C—H⋯O hydrogen bonds and C=O⋯π inter­actions; π–π inter­actions are also present. With respective average deviations from planarity of 0.003 (2) and 0.002 (1) Å, the xanthone and ester fragments are oriented at an angle of 2.8 (2)° with respect to each other. The mean planes of the xanthone skeleton lie either parallel to each other or are inclined at an angle of 85.5 (2)° in the crystal structure.

## Related literature

For general background and uses of xanthones, see: Chen *et al.* (1993 ▶); Denisova-Dyatlova & Glyzin (1982 ▶); Fukai *et al.* (2005 ▶); Gopalakrishnan *et al.* (1997 ▶); Ignatushchenko *et al.* (2000 ▶); Ito *et al.* (2003 ▶); Librowski *et al.* (2005 ▶); Pfister *et al.* (1972 ▶, 1980 ▶). For related structures, see: Evans *et al.* (2004 ▶); Shi *et al.* (2004 ▶); Macias *et al.* (2001 ▶). For synthesis, see: Geertsema *et al.* (2006 ▶). For background to the various types of inter­molecular inter­actions, see: Bianchi *et al.* (2004 ▶); Steiner (1999 ▶) Santos-Contreras *et al.* (2007 ▶); Hunter & Sanders (1990 ▶). For analysis of inter­molecular inter­actions, see: Spek (2003 ▶). 
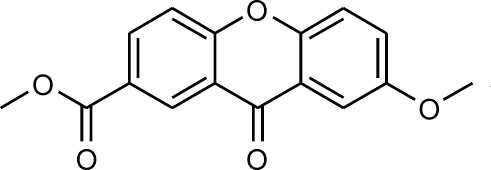


## Experimental

### 

#### Crystal data

C_16_H_12_O_5_
*M* *_r_* = 284.26
Monoclinic, 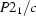

*a* = 4.7709 (4) Å
*b* = 10.5375 (8) Å
*c* = 26.7854 (19) Å
β = 93.266 (7)°
*V* = 1344.40 (18) Å^3^
*Z* = 4
Mo *K*α radiation
μ = 0.11 mm^−1^
*T* = 295 (2) K
0.20 × 0.04 × 0.04 mm

#### Data collection

Oxford Diffraction Ruby CCD diffractometer
Absorption correction: multi-scan (*CrysAlis RED*; Oxford Diffraction, 2008 ▶) *T*_min_ = 0.994, *T*_max_ = 0.997
23842 measured reflections
2366 independent reflections
1051 reflections with *I* > 2σ(*I*)
*R*_int_ = 0.086

#### Refinement

*R*[*F* ^2^ > 2σ(*F* ^2^)] = 0.038
*wR*(*F* ^2^) = 0.092
*S* = 0.81
2366 reflections
193 parameters
H-atom parameters constrained
Δρ_max_ = 0.13 e Å^−3^
Δρ_min_ = −0.14 e Å^−3^

### 

Data collection: *CrysAlis CCD* (Oxford Diffraction, 2008 ▶); cell refinement: *CrysAlis RED* (Oxford Diffraction, 2008 ▶); data reduction: *CrysAlis RED*; program(s) used to solve structure: *SHELXS97* (Sheldrick, 2008 ▶); program(s) used to refine structure: *SHELXL97* (Sheldrick, 2008 ▶); molecular graphics: *ORTEPII* (Johnson, 1976 ▶); software used to prepare material for publication: *SHELXL97* and *PLATON* (Spek, 2003 ▶).

## Supplementary Material

Crystal structure: contains datablocks I, global. DOI: 10.1107/S1600536809003602/xu2476sup1.cif

Structure factors: contains datablocks I. DOI: 10.1107/S1600536809003602/xu2476Isup2.hkl

Additional supplementary materials:  crystallographic information; 3D view; checkCIF report

## Figures and Tables

**Table 1:** Hydrogen-bond geometry (Å, °)

*D*—H⋯*A*	*D*—H	H⋯*A*	*D*⋯*A*	*D*—H⋯*A*
C3—H3⋯O16^i^	0.93	2.54	3.362 (3)	147
C20—H20*A*⋯O21^ii^	0.96	2.50	3.454 (3)	173

Symmetry codes: (i) 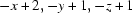; (ii) 
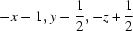
.

**Table 2:** π–π interactions (Å,°)

*CgI*	*CgJ*	*Cg*⋯*Cg*	Dihedral angle	Interplanar distance	Offset
*A*	*C*^iii^	3.549 (1)	0.8	3.420 (1)	1.068 (1)
*B*	*A*^iii^	3.583 (1)	0.1	3.454 (1)	0.953 (1)
*B*	*C*^iii^	3.772 (1)	0.8	3.455 (1)	1.525 (1)

Symmetry code: (iii) 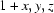. *CgA*, *CgB* and *CgC* are the centroids of the C9/O10/C11–C14, C1–C4/C12/C11 and C5–C8/C13/C14 rings, respectively. The dihedral angle is that between the planes of the rings *CgI* and *CgJ*. The interplanar distance is the perpendicular distance of *CgI* from ring *J*. The offset is the perpendicular distance of ring *I* from ring *J*.

**Table 3:** C—O⋯π interactions (Å,°)

*X*	*I*	*J*	*I*⋯*J*	*X*⋯*J*	*X*—*I*⋯*J*
C15	O16	*CgB*^iii^	3.564 (2)	3.689 (2)	86.4 (1)

Symmetry code: (iii) 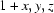. *CgB* is the centroid of the C1–C4/C12/C11 ring.

**Fig. 1.**
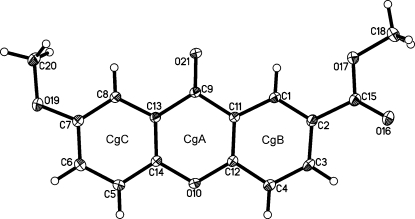
The molecular structure of the title compound showing the atom-labeling scheme. Displacement ellipsoids are drawn at the 25% probability level and H atoms are shown as small spheres of arbitrary radius. CgA, CgB and CgC denote the ring centroids.

**Fig. 2.**
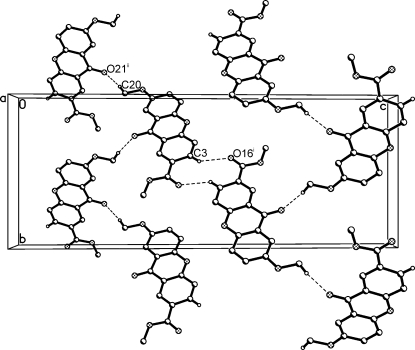
The arrangement of the molecules in the crystal structure viewed approximately along a axis. The C—H···O interactions are represented by dashed lines. H atoms not involved in the interactions have been omitted. [Symmetry codes: (i) 2 - x, 1 - y, 1 - z; (ii) -1 - x, -1/2 + y, 1/2 - z.]

**Fig. 3.**
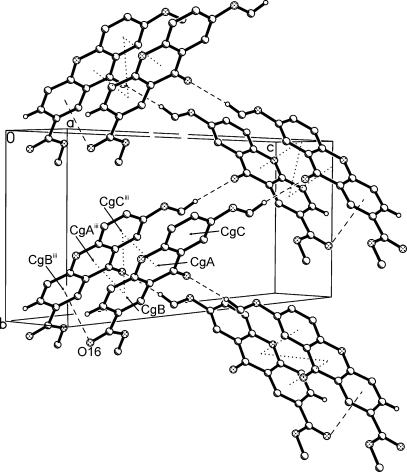
The arrangement of the molecules in the crystal structure viewed approximately along a axis. The C—H···O and C—O···π interactions are represented by dashed lines and the π–π interactions are represented by dotted lines. H atoms not involved in the interactions have been omitted. [Symmetry codes: (iii) 1 + x, y, z.]

## supplementary crystallographic information

### Comment

Xanthones represent a structurally diverse group of natural products with a broad range of biological activities. The unsubstituted xanthones have not been discovered in nature but its numerous derivatives have been isolated from representatives of higher plants, lichens, and lower fungi (Denisova-Dyatlova & Glyzin, 1982). Many naturally occurring xanthones as well as their synthetic derivatives described in numerous scientific publications exploit wide spectrum of biological activities: anti-allergic (Pfister *et al.*, 1972), anti-inflammatory (Librowski *et al.*, 2005), antitumor (Ito *et al.*, 2003), antimicrobial (Fukai *et al.*, 2005), cardiovascular (Chen *et al.*, 1993), antimalarial (Gopalakrishnan *et al.*, 1997) and antifungal activity (Ignatushchenko *et al.*, 2000). The biological activity and the features responsible for the activity of xanthones largely depends on their structures. It is know that the 7-substituted xanthone-2-carboxylic acids and their esters show anti-allergic activity, which depends on the substituted groups (Pfister *et al.*, 1980).

In the molecule of the title compound (Fig. 1) the bond lengths and angles characterizing the geometry of the xanthone skeleton are typical for this group compounds (Evans *et al.*, 2004; Shi *et al.*, 2004; Macias *et al.*, 2001).

With respective average deviations from planarity of 0.003 (2) and 0.002 (1) Å, the xanthone and ester fragment are oriented at 2.8 (2)° to each other. The methoxy group lies nearly in the mean plane of the xanthone skeleton; the dihedral angles between the mean planes xanthone skeleton and delineated by atoms C7/O19/C20 are equal 0.7 (2)°. The mean planes of the xanthone skeleton lie either parallel or are inclined at an angle of 85.5 (2)° in the lattice.

In the crystal structure, weak intermolecular C—H···O hydrogen bonds (Table 1, Fig. 2) link the molecules, forming layers. The central ring A and the lateral rings B and C are involved in multidirectional π–π interactions and link layers between themselves (Table 2, Fig. 3). The O16(carboxyl) atom is involved in weak C—O···π interactions directed toward the lateral aromatic ring (ring B) (Table 3, Fig. 3).

All the interactions demonstrated were found by *PLATON* (Spek, 2003). The C—H···O (Bianchi *et al.*, 2004; Steiner, 1999) interactions exhibit a hydrogen-bond-type nature. The C—O(carbonyl)···π interactions (Santos-Contreras *et al.*, 2007), and also π–π interactions (Hunter & Sanders, 1990) should be of an attractive nature.

### Experimental

7-Methoxy-9-oxo-9*H*-xanthene-2-carboxylatic acid methyl ester was synthesized by three steps. First, in a nucleophilic substitution of 4-methoxyphenol and 4-bromoisophthalic acid, to yield 4-(4-methoxyphenoxy)isophthalic acid, by refluxing 45 min in *N*,*N*-dimethylformamide with potassium carbonate, sodium iodide and activated Cu-bronze. In the next reaction, called intramolecular Friedel–Crafts acylation was synthesized 7-methoxy-9-oxo-9*H*-xanthene-2-carboxylatic acid (Geertsema *et al.*, 2006). In last step 7-methoxy-9-oxo-9*H*-xanthene-2-carboxylatic acid was esterified with methanol by refluxing in thionyl chloride in 45 min and then treated with mixture of methanol and triethylamine in room temperature by 12 h with catalytic amount of 4-dimethylaminopyridine (DMAP). The crude product was dissolved in small amount of anhydrous methanol to obtain single crystals suitable for X-ray analysis by slow evaporation of methanol solution at 298 K.

### Refinement

All H atoms were positioned geometrically and refined using a riding model, with C—H = 0.93 Å and *U*_iso_(H) = 1.2*U*_eq_(C) for aromatic, and with C—H = 0.96 Å and *U*_iso_(H) = 1.5*U*_eq_(C) for methyl groups.

### Crystal data

**Table:**

C_16_H_12_O_5_	*F*(000) = 592.0
*M**_r_* = 284.26	*D*_x_ = 1.404 Mg m^−^^3^
Monoclinic, *P*2_1_/*c*	Mo *K*α radiation, λ = 0.71073 Å
Hall symbol: -P 2ybc	Cell parameters from 2126 reflections
*a* = 4.7709 (4) Å	θ = 3.0–25.0°
*b* = 10.5375 (8) Å	µ = 0.11 mm^−^^1^
*c* = 26.7854 (19) Å	*T* = 295 K
β = 93.266 (7)°	Needle, white
*V* = 1344.40 (18) Å^3^	0.2 × 0.04 × 0.04 mm
*Z* = 4	

### Data collection

**Table:**

Oxford Diffraction Ruby CCD diffractometer	2366 independent reflections
Radiation source: Enhance (Mo) X-ray Source	1051 reflections with *I* > 2σ(*I*)
graphite	*R*_int_ = 0.086
Detector resolution: 10.4002 pixels mm^-1^	θ_max_ = 25.0°, θ_min_ = 3.0°
ω scans	*h* = −5→5
Absorption correction: multi-scan (*CrysAlis RED*; Oxford Diffraction, 2008)	*k* = −12→12
*T*_min_ = 0.994, *T*_max_ = 0.997	*l* = −31→31
23842 measured reflections	

### Refinement

**Table:**

Refinement on *F*^2^	Primary atom site location: structure-invariant direct methods
Least-squares matrix: full	Secondary atom site location: difference Fourier map
*R*[*F*^2^ > 2σ(*F*^2^)] = 0.038	Hydrogen site location: inferred from neighbouring sites
*wR*(*F*^2^) = 0.092	H-atom parameters constrained
*S* = 0.81	*w* = 1/[σ^2^(*F*_o_^2^) + (0.0471*P*)^2^] where *P* = (*F*_o_^2^ + 2*F*_c_^2^)/3
2366 reflections	(Δ/σ)_max_ = 0.001
193 parameters	Δρ_max_ = 0.13 e Å^−^^3^
0 restraints	Δρ_min_ = −0.13 e Å^−^^3^

### Special details

**Table:**

Experimental. CrysAlis RED, Version 1.171.32.15 (Oxford Diffraction Ltd., 2008) Empirical absorption correction using spherical harmonics, implemented in SCALE3 ABSPACK scaling algorithm.
Geometry. All e.s.d.'s (except the e.s.d. in the dihedral angle between two l.s. planes) are estimated using the full covariance matrix. The cell e.s.d.'s are taken into account individually in the estimation of e.s.d.'s in distances, angles and torsion angles; correlations between e.s.d.'s in cell parameters are only used when they are defined by crystal symmetry. An approximate (isotropic) treatment of cell e.s.d.'s is used for estimating e.s.d.'s involving l.s. planes.
Refinement. Refinement of *F*^2^ against ALL reflections. The weighted *R*-factor *wR* and goodness of fit *S* are based on *F*^2^, conventional *R*-factors *R* are based on *F*, with *F* set to zero for negative *F*^2^. The threshold expression of *F*^2^ > σ(*F*^2^) is used only for calculating *R*-factors(gt) *etc*. and is not relevant to the choice of reflections for refinement. *R*-factors based on *F*^2^ are statistically about twice as large as those based on *F*, and *R*- factors based on ALL data will be even larger.

### Fractional atomic coordinates and isotropic or equivalent isotropic displacement parameters (Å^2^)

**Table:**

	*x*	*y*	*z*	*U*_iso_*/*U*_eq_	
C1	0.4869 (4)	0.3741 (2)	0.37390 (8)	0.0507 (6)	
H1	0.4950	0.4005	0.3409	0.061*	
C2	0.6568 (4)	0.4321 (2)	0.41017 (8)	0.0531 (6)	
C3	0.6434 (5)	0.3912 (2)	0.45962 (9)	0.0678 (7)	
H3	0.7584	0.4289	0.4846	0.081*	
C4	0.4634 (5)	0.2964 (3)	0.47187 (9)	0.0735 (7)	
H4	0.4548	0.2703	0.5049	0.088*	
C5	−0.2272 (5)	−0.0083 (2)	0.43087 (9)	0.0742 (7)	
H5	−0.2221	−0.0287	0.4647	0.089*	
C6	−0.4064 (5)	−0.0705 (2)	0.39752 (9)	0.0723 (7)	
H6	−0.5229	−0.1337	0.4089	0.087*	
C7	−0.4169 (4)	−0.0408 (2)	0.34712 (9)	0.0570 (6)	
C8	−0.2448 (4)	0.0520 (2)	0.33022 (8)	0.0506 (6)	
H8	−0.2514	0.0723	0.2964	0.061*	
C9	0.1240 (4)	0.2157 (2)	0.34601 (8)	0.0485 (5)	
O10	0.1221 (3)	0.14529 (15)	0.44927 (5)	0.0685 (5)	
C11	0.3026 (4)	0.2770 (2)	0.38514 (7)	0.0469 (5)	
C12	0.2945 (4)	0.2399 (2)	0.43445 (8)	0.0567 (6)	
C13	−0.0592 (4)	0.1163 (2)	0.36375 (7)	0.0459 (5)	
C14	−0.0538 (4)	0.0853 (2)	0.41369 (8)	0.0567 (6)	
C15	0.8493 (5)	0.5368 (2)	0.39863 (10)	0.0616 (6)	
O16	0.9949 (4)	0.59309 (17)	0.42912 (7)	0.0897 (6)	
O17	0.8478 (3)	0.56064 (15)	0.34987 (6)	0.0748 (5)	
C18	1.0307 (5)	0.6610 (2)	0.33454 (10)	0.0877 (8)	
H18A	0.9931	0.6783	0.2996	0.132*	
H18B	1.2228	0.6352	0.3403	0.132*	
H18C	0.9978	0.7362	0.3536	0.132*	
O19	−0.6050 (3)	−0.10926 (15)	0.31785 (6)	0.0734 (5)	
C20	−0.6235 (5)	−0.0814 (3)	0.26581 (9)	0.0837 (8)	
H20A	−0.7623	−0.1351	0.2493	0.126*	
H20B	−0.4446	−0.0962	0.2522	0.126*	
H20C	−0.6760	0.0058	0.2609	0.126*	
O21	0.1291 (3)	0.24526 (14)	0.30176 (5)	0.0657 (5)	

### Atomic displacement parameters (Å^2^)

**Table:**

	*U*^11^	*U*^22^	*U*^33^	*U*^12^	*U*^13^	*U*^23^
C1	0.0541 (13)	0.0547 (16)	0.0427 (13)	0.0009 (12)	−0.0024 (11)	−0.0013 (11)
C2	0.0517 (13)	0.0553 (16)	0.0518 (15)	−0.0049 (11)	−0.0025 (11)	−0.0058 (12)
C3	0.0741 (15)	0.0794 (19)	0.0481 (16)	−0.0117 (14)	−0.0139 (11)	−0.0116 (14)
C4	0.0851 (17)	0.087 (2)	0.0471 (14)	−0.0246 (16)	−0.0071 (13)	0.0017 (14)
C5	0.0855 (16)	0.084 (2)	0.0519 (15)	−0.0292 (15)	−0.0047 (13)	0.0166 (14)
C6	0.0777 (16)	0.0703 (19)	0.0681 (19)	−0.0229 (14)	−0.0016 (14)	0.0133 (15)
C7	0.0565 (13)	0.0599 (16)	0.0535 (15)	−0.0049 (13)	−0.0053 (11)	0.0004 (13)
C8	0.0532 (12)	0.0517 (15)	0.0464 (13)	−0.0031 (11)	−0.0022 (11)	−0.0001 (11)
C9	0.0491 (13)	0.0508 (15)	0.0449 (14)	0.0006 (11)	−0.0036 (11)	0.0031 (12)
O10	0.0800 (10)	0.0808 (13)	0.0429 (9)	−0.0254 (9)	−0.0104 (8)	0.0102 (8)
C11	0.0475 (12)	0.0498 (15)	0.0427 (13)	−0.0023 (11)	−0.0027 (10)	0.0012 (11)
C12	0.0584 (13)	0.0629 (17)	0.0476 (14)	−0.0139 (13)	−0.0063 (11)	0.0007 (12)
C13	0.0470 (12)	0.0466 (14)	0.0436 (14)	0.0005 (11)	−0.0022 (10)	0.0020 (11)
C14	0.0617 (14)	0.0596 (17)	0.0473 (15)	−0.0124 (12)	−0.0087 (11)	0.0037 (12)
C15	0.0620 (15)	0.0620 (18)	0.0598 (17)	−0.0037 (13)	−0.0044 (12)	−0.0063 (15)
O16	0.1020 (13)	0.0906 (14)	0.0742 (12)	−0.0367 (11)	−0.0143 (10)	−0.0106 (11)
O17	0.0865 (11)	0.0729 (13)	0.0641 (12)	−0.0294 (10)	−0.0039 (9)	0.0052 (9)
C18	0.0935 (18)	0.078 (2)	0.092 (2)	−0.0274 (16)	0.0074 (15)	0.0129 (16)
O19	0.0772 (10)	0.0744 (12)	0.0671 (12)	−0.0265 (9)	−0.0081 (8)	−0.0049 (9)
C20	0.0931 (18)	0.102 (2)	0.0549 (17)	−0.0282 (16)	−0.0058 (13)	−0.0135 (15)
O21	0.0744 (10)	0.0781 (12)	0.0432 (9)	−0.0200 (8)	−0.0084 (7)	0.0105 (8)

### Geometric parameters (Å, °)

**Table:**

C1—C2	1.373 (3)	C9—O21	1.227 (2)
C1—C11	1.394 (3)	C9—C13	1.461 (3)
C1—H1	0.9300	C9—C11	1.462 (3)
C2—C3	1.398 (3)	O10—C12	1.366 (2)
C2—C15	1.480 (3)	O10—C14	1.386 (2)
C3—C4	1.369 (3)	C11—C12	1.380 (3)
C3—H3	0.9300	C13—C14	1.376 (3)
C4—C12	1.384 (3)	C15—O16	1.199 (2)
C4—H4	0.9300	C15—O17	1.330 (3)
C5—C6	1.368 (3)	O17—C18	1.446 (3)
C5—C14	1.383 (3)	C18—H18A	0.9600
C5—H5	0.9300	C18—H18B	0.9600
C6—C7	1.384 (3)	C18—H18C	0.9600
C6—H6	0.9300	O19—C20	1.422 (3)
C7—O19	1.364 (2)	C20—H20A	0.9600
C7—C8	1.370 (3)	C20—H20B	0.9600
C8—C13	1.400 (3)	C20—H20C	0.9600
C8—H8	0.9300		
			
C2—C1—C11	121.9 (2)	C12—C11—C9	120.9 (2)
C2—C1—H1	119.1	C1—C11—C9	121.22 (19)
C11—C1—H1	119.1	O10—C12—C11	122.33 (19)
C1—C2—C3	118.4 (2)	O10—C12—C4	116.0 (2)
C1—C2—C15	122.2 (2)	C11—C12—C4	121.6 (2)
C3—C2—C15	119.4 (2)	C14—C13—C8	119.0 (2)
C4—C3—C2	121.0 (2)	C14—C13—C9	120.5 (2)
C4—C3—H3	119.5	C8—C13—C9	120.44 (19)
C2—C3—H3	119.5	C13—C14—C5	121.0 (2)
C3—C4—C12	119.2 (2)	C13—C14—O10	122.5 (2)
C3—C4—H4	120.4	C5—C14—O10	116.5 (2)
C12—C4—H4	120.4	O16—C15—O17	123.1 (2)
C6—C5—C14	119.2 (2)	O16—C15—C2	124.7 (2)
C6—C5—H5	120.4	O17—C15—C2	112.2 (2)
C14—C5—H5	120.4	C15—O17—C18	116.59 (19)
C5—C6—C7	121.0 (2)	O17—C18—H18A	109.5
C5—C6—H6	119.5	O17—C18—H18B	109.5
C7—C6—H6	119.5	H18A—C18—H18B	109.5
O19—C7—C8	125.1 (2)	O17—C18—H18C	109.5
O19—C7—C6	115.3 (2)	H18A—C18—H18C	109.5
C8—C7—C6	119.6 (2)	H18B—C18—H18C	109.5
C7—C8—C13	120.2 (2)	C7—O19—C20	117.16 (17)
C7—C8—H8	119.9	O19—C20—H20A	109.5
C13—C8—H8	119.9	O19—C20—H20B	109.5
O21—C9—C13	122.78 (19)	H20A—C20—H20B	109.5
O21—C9—C11	122.5 (2)	O19—C20—H20C	109.5
C13—C9—C11	114.73 (19)	H20A—C20—H20C	109.5
C12—O10—C14	118.97 (16)	H20B—C20—H20C	109.5
C12—C11—C1	117.8 (2)		
			
C11—C1—C2—C3	0.3 (3)	C3—C4—C12—C11	−0.1 (4)
C11—C1—C2—C15	−178.91 (19)	C7—C8—C13—C14	−0.3 (3)
C1—C2—C3—C4	−0.6 (3)	C7—C8—C13—C9	−179.76 (19)
C15—C2—C3—C4	178.6 (2)	O21—C9—C13—C14	178.94 (19)
C2—C3—C4—C12	0.6 (4)	C11—C9—C13—C14	−0.7 (3)
C14—C5—C6—C7	−0.2 (4)	O21—C9—C13—C8	−1.6 (3)
C5—C6—C7—O19	−179.7 (2)	C11—C9—C13—C8	178.67 (18)
C5—C6—C7—C8	0.2 (4)	C8—C13—C14—C5	0.3 (3)
O19—C7—C8—C13	−179.99 (19)	C9—C13—C14—C5	179.7 (2)
C6—C7—C8—C13	0.1 (3)	C8—C13—C14—O10	−179.42 (18)
C2—C1—C11—C12	0.1 (3)	C9—C13—C14—O10	0.0 (3)
C2—C1—C11—C9	−179.66 (19)	C6—C5—C14—C13	−0.1 (4)
O21—C9—C11—C12	−179.0 (2)	C6—C5—C14—O10	179.7 (2)
C13—C9—C11—C12	0.7 (3)	C12—O10—C14—C13	0.8 (3)
O21—C9—C11—C1	0.8 (3)	C12—O10—C14—C5	−178.9 (2)
C13—C9—C11—C1	−179.53 (17)	C1—C2—C15—O16	177.5 (2)
C14—O10—C12—C11	−0.8 (3)	C3—C2—C15—O16	−1.7 (4)
C14—O10—C12—C4	179.6 (2)	C1—C2—C15—O17	−3.8 (3)
C1—C11—C12—O10	−179.70 (17)	C3—C2—C15—O17	177.05 (19)
C9—C11—C12—O10	0.1 (3)	O16—C15—O17—C18	−0.8 (3)
C1—C11—C12—C4	−0.2 (3)	C2—C15—O17—C18	−179.59 (18)
C9—C11—C12—C4	179.6 (2)	C8—C7—O19—C20	−0.2 (3)
C3—C4—C12—O10	179.4 (2)	C6—C7—O19—C20	179.7 (2)

### Hydrogen-bond geometry (Å, °)

**Table:**

*D*—H···*A*	*D*—H	H···*A*	*D*···*A*	*D*—H···*A*
C3—H3···O16^i^	0.93	2.54	3.362 (3)	147
C20—H20A···O21^ii^	0.96	2.50	3.454 (3)	173

Symmetry codes: (i) −*x*+2, −*y*+1, −*z*+1; (ii) −*x*−1, *y*−1/2, −*z*+1/2.

### Table 2 π–π interactions (Å,°).

**Table:**

CgI	CgJ	Cg···Cg	Dihedral angle	Interplanar distance	Offset
A	C^iii^	3.549 (1)	0.8	3.420 (1)	1.068 (1)
B	A^iii^	3.583 (1)	0.1	3.454 (1)	0.953 (1)
B	C^iii^	3.772 (1)	0.8	3.455 (1)	1.525 (1)

Symmetry code: (iii) 1 + x, y, z. CgA, CgB and CgC are the centroids of the C9/O10/C11–C14, C1–C4/C12/C11 and C5–C8/C13/C14 rings, respectively. The dihedral angle is that between the planes of the rings CgI and CgJ. The interplanar distance is the perpendicular distance of CgI from ring J. The offset is the perpendicular distance of ring I from ring J.

### Table 3 C—O···π interactions (Å,°)

**Table:**

X	I	J	I···J	X···J	X-I···J
C15	O16	CgB^iii^	3.564 (2)	3.689 (2)	86.4 (1)

Symmetry codes: (iii) 1 + x, y, z.Notes: CgB is the centroid of the C1–C4/C12/C11 ring.

## References

[bb1] Bianchi, R., Forni, A. & Pilati, T. (2004). *Acta Cryst.* B**60**, 559–568. 10.1107/S0108768104014557 15367791

[bb2] Chen, I. J., Liou, S. J., Liou, S. S. & Lin, C. N. (1993). *Gen. Pharmacol.* **24**, 1425–1433. 10.1016/0306-3623(93)90430-6 7906662

[bb3] Denisova-Dyatlova, O. A. & Glyzin, V. I. (1982). *Russ. Chem. Rev.* **51**, 1753–1774.

[bb4] Evans, I. R., Howard, J. A. K., Šavikin-Fodulović, K. & Menković, N. (2004). *Acta Cryst.* E**60**, o1557–o1559.

[bb5] Fukai, T., Oku, Y., Hou, A. J., Yonekawa, Y. M. & Terada, S. (2005). *Phytomedicine*, **12**, 510–513. 10.1016/j.phymed.2004.03.010 16008130

[bb6] Geertsema, E. M., Hoen, R., Meetsma, A. & Feringa, B. L. (2006). *Eur. J. Org. Chem.* **16**, 3596–3605.

[bb7] Gopalakrishnan, G., Banumathi, B. & Suresh, G. (1997). *J. Nat. Prod.* **60**, 519–524. 10.1021/np970165u 9213587

[bb8] Hunter, C. A. & Sanders, J. K. M. (1990). *J. Am. Chem. Soc.* **112**, 5525–5534.

[bb9] Ignatushchenko, M. V., Winter, R. W. & Riscoe, M. (2000). *Am. J. Trop. Med. Hyg.* **62**, 2000, 77–81. 10.4269/ajtmh.2000.62.77 10761728

[bb10] Ito, C., Itoigawa, M., Takakura, T., Ruangrungsi, N., Enjo, F., Tokuda, H., Nishino, H. & Furukawa, H. (2003). *J. Nat. Prod.* **66**, 200–205. 10.1021/np020290s 12608849

[bb11] Johnson, C. K. (1976). *ORTEPII* Report ORNL-5138. Oak Ridge National Laboratory, Tennessee, USA.

[bb12] Librowski, T., Czarnecki, R., Czekaj, T. & Marona, H. (2005). *Medicina (Kaunas)*, **41**, 54–58. 15687751

[bb13] Macias, M., Gamboa, A., Ulloa, M., Toscano, R. A. & Mata, R. (2001). *Phytochemistry*, **58**, 751–758. 10.1016/s0031-9422(01)00278-3 11672740

[bb14] Oxford Diffraction (2008). *CrysAlis CCD* and *CrysAlis RED* Oxford Diffraction Ltd, Abingdon, Oxfordshire, England.

[bb15] Pfister, J. R., Ferraresi, R. W., Harrison, I. T., Rooks, W. H., Roszkowski, A. P., Van Horn, A. & Fried, J. H. (1972). *J. Med. Chem.* **15**, 1032–1035. 10.1021/jm00280a010 5069771

[bb16] Pfister, J. R., Weymann, W. E., Mahoney, J. M. & Waterbury, L. D. (1980). *J. Med. Chem.* **23**, 1264–1267. 10.1021/jm00185a027 6779009

[bb17] Santos-Contreras, R. J., Martínez-Martínez, F. J., García-Báez, E. V., Padilla-Martínez, I. I., Peraza, A. L. & Höpfl, H. (2007). *Acta Cryst.* C**63**, o239–o242. 10.1107/S0108270107008712 17413237

[bb18] Sheldrick, G. M. (2008). *Acta Cryst.* A**64**, 112–122. 10.1107/S0108767307043930 18156677

[bb19] Shi, G.-F., Lu, R.-H., Yang, Y.-S., Li, C.-L., Yang, A.-M. & Cai, L.-X. (2004). *Acta Cryst.* E**60**, o878–o880.

[bb20] Spek, A. L. (2003). *J. Appl. Cryst.* **36**, 7–13.

[bb21] Steiner, T. (1999). *Chem. Commun.* pp. 313–314.

